# Surgical treatment of a persistent right aortic arch with concurrent patent ductus arteriosus in a 4‐month‐old German shepherd dog

**DOI:** 10.1002/vms3.1164

**Published:** 2023-05-27

**Authors:** Marco T. Duguay, Meagan A. Walker, Justyna Ostrowska, Katie L. Hoddinott

**Affiliations:** ^1^ Atlantic Veterinary College Department of Companion Animal Surgery University of Prince Edward Island Charlottetown Canada; ^2^ Vet‐CT Teleradiology St John's Innovation Centre Cambridge UK

**Keywords:** persistent right aortic arch, patent ductus arteriosus, regurgitation, thoracotomy

## Abstract

A 4‐month‐old intact female German shepherd dog was presented with a history of postprandial regurgitation, a palpably distended cervical oesophagus after eating, and poor weight gain despite a ravenous appetite. Computed tomography angiography, esophagoscopy and echocardiography identified a persistent right aortic arch with a concurrent patent ductus arteriosus (PDA) causing extraluminal oesophageal compression leading to marked segmental megaoesophagus. A heart murmur was not detectable. A left lateral thoracotomy was performed to ligate and transect the PDA without complication. The dog was discharged with mild aspiration pneumonia which resolved with antimicrobial therapy. Twelve months post‐surgery the owners reported no regurgitation.

## Graphical abstract

This is the first case report of a dog with simultaneous PRAA and PDA, lacking an audible heart murmur. Use of advanced imaging is key in characterizing vascular anomalies and determining best surgical approaches. A PDA cannot be definitively excluded due to lack of heart murmur in the presence of a PRAA.

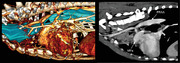

## 1 INTRODUCTION

The ductus arteriosus (DA) is a foetal vessel that connects the permanent arch of the aorta to the pulmonary artery. The DA allows blood to shunt from the pulmonary artery to the aorta, allowing maternal oxygenated blood to bypass the foetal lungs and flow directly into circulation. Shortly after birth, the DA closes, but its attachments to the aorta and pulmonary artery remain as a fibrous structure known as the ligamentum arteriosum (LA). Occasionally, the DA will not close during development and a patent vessel connecting the aorta and the pulmonary artery remains, known as a patent ductus arteriosus (PDA) (Buchanan, 2003).

Persistent right aortic arch (PRAA) is a vascular anomaly caused by the failure of regression of the forth right aortic arch. This results in a right paramedian position of the permanent arch of the aorta. With a left LA, the thoracic trachea and oesophagus become compressed by a vascular ring formed by the permanent arch of the aorta and the LA, resulting in cranial oesophageal dilation. PRAA with a left LA is the most common clinical vascular ring anomaly in canines, representing 95% of clinical cases (Buchanan, 2004). Surgical treatment, via transection of the LA, is indicated in these patients. It has been estimated that PDA occurs concurrently in only 10%–14% of animals with PRAA (Buchanan, 2004; Schorn et al., 2021; vanGrundy, 1989).

## 2 CASE DESCRIPTION

A 4‐month‐old intact female German shepherd dog was presented with a history of daily postprandial regurgitation, a palpably distended cervical oesophagus after eating and poor weight gain despite a ravenous appetite. Clinical signs had been present since adoption at 7 weeks of age. The body weight at the time of adoption was 2.3 kg; significantly lower than other littermates.

A lateral thoracic radiograph provided by the referring veterinarian revealed marked oesophageal dilation cranial to the base of the heart, with a food bolus present within the lumen and a ventrally displaced trachea. At the time of presentation, the dog was receiving metoclopramide 0.21 mg/kg body weight (BW, PO; q8) (Apo‐Metoclop; Apotex Inc., Weston, Ontario). The dog was being elevated for 15 min after being fed kibble from a non‐elevated slow feeder. Mild improvement in frequency of regurgitation was noted following implementation of these changes.

Upon presentation, the dog was bright, alert and responsive with a body weight of 11.9 kg, and a body condition score of 3/9. All vital parameters were within normal limits. Cardiac auscultation revealed a normal rhythm and no detectable murmur. Normal bronchovesicular lungs sounds were appreciated in all fields, with normal respiratory effort. A 3 cm tubular soft‐tissue structure, presumed to be the oesophagus, was palpated along the ventral aspect of the cervical region. The remainder of the physical exam was unremarkable.

Based on the dog's clinical signs, age of onset and the preliminary radiographic findings, the primary differential was a vascular ring anomaly, with a PRAA being considered most likely. Other differential diagnoses to consider based on clinical signs and age of onset included idiopathic primary megaoesophagus, hiatal hernia and gastroesophageal intussusception.

Diagnostic evaluation included complete blood count, serum biochemistry, thoracic computed tomography angiography (CTA), esophagoscopy and echocardiography. Complete blood count and serum biochemistry revealed minor changes associated with decrease muscle mass and food intake (creatinine: 47 μmol/L [54–122 μmol/L], magnesium: 0.65 mmol/L [0.70–1.16 mmol/L], total protein: 51 g/L [56–71 g/L], albumin: 26 g/L [30–36 g/L]) and changes attributed to the dog's young age (phosphorus: 2.52 mmol/L [0.84–1.83 mmol/L], alkaline phosphatase: 150 U/L [18–113 U/L], creatine kinase: 298 U/L [44–249 U/L], red blood cells: 5.12 × 10^12^/L [5.7–8.4 × 10^12^/L], haemoglobin: 115 g/L [135–198 g/L] and haematocrit: 0.337 L/L [0.40–0.56 L/L]).

**FIGURE 1:**
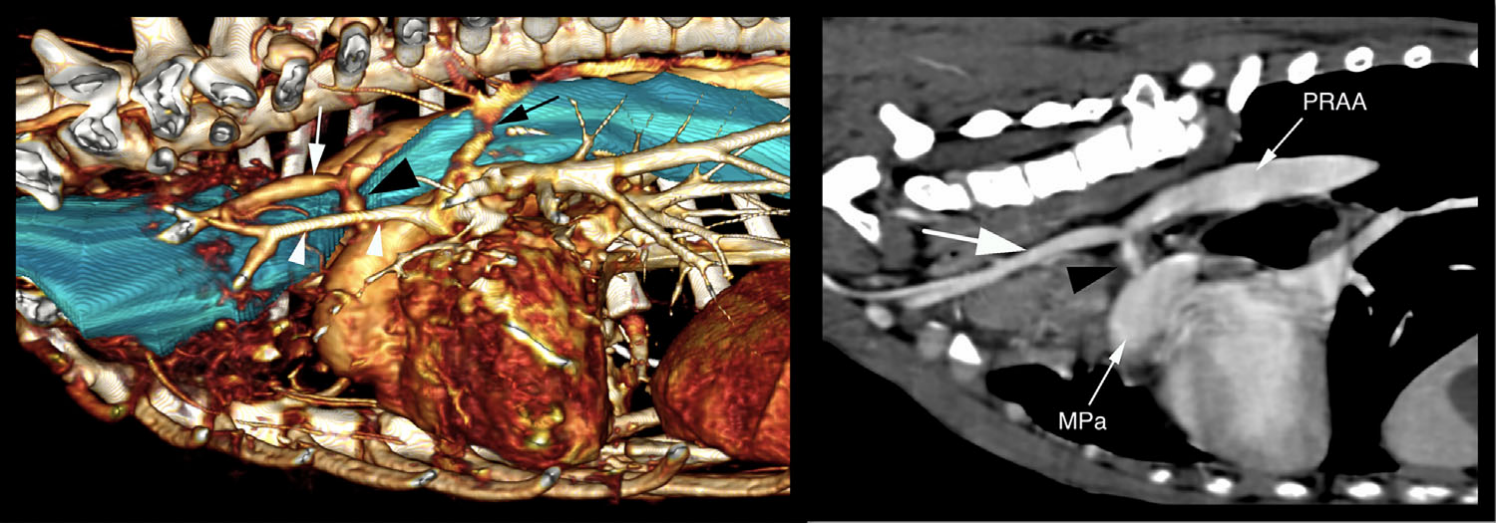
Three‐dimensional (3D) volume rendered image of the heart and major vasculature (left hand image) and sagittal multiplanar reconstruction at the level of the patent ductus arteriosus (PDA) (right hand image). The oesophagus is coloured in turquoise. PRAA – persistent right aortic arch; MPa – main pulmonary artery; black arrowhead – PDA; large white arrow – aberrant left subclavian artery, white arrowheads – anomalous left vertebral vein and black arrow – left azygos vein.

The dog underwent general anaesthesia for thoracic CTA and esophagoscopy. CTA revealed multiple vascular anomalies, including a PRAA, PDA, aberrant left subclavian artery, anomalous left vertebral vein and a persistent left azygos vein (Figure 1). The most clinically relevant vascular anomalies were the PRAA and the PDA. The aortic arch was visualized extending paramedian on the right side of the thorax causing leftward deviation and narrowing of the thoracic trachea at the third intercostal space PRAA. At the fourth intercostal space, the trachea was noted to be surrounded by a vascular structure connecting the main pulmonary artery to the origin of the aberrant left subclavian artery and descending aorta PDA. No dilation of the main pulmonary artery or aorta was noted. An echocardiogram confirmed a left to right shunting PDA with no secondary cardiovascular changes.

Esophagoscopy (Pentax EG‐29901 2.8; Pentax Medical, Akishima‐shi, Tokyo,) performed immediately post CTA confirmed dilation of the oesophagus cranial to the base of the heart. Oesophageal compression was present near the base of the heart and an external pulse was visualized over the right side of the oesophagus corresponding with the right sided aortic arch. Passing the endoscope through the lumen of the compressed region revealed a dilated caudal oesophagus, although markedly less dilated than the cranial portion. These findings were consistent with the PRAA identified on CTA.

With the combination of a PRAA and a PDA causing extraluminal oesophageal compression, ligation and division of the PDA via left lateral thoracotomy was recommended. The dog was premedicated with fentanyl (5 μg/kg BW, IV) (Fentanyl; McKesson Canada, Saint‐Laurent, Quebec) and midazolam (0.1 mg/kg BW, IV) (Midazolam; Sandoz Canada Inc, Quebec, Canada). Profound sedation was achieved, and an induction agent was not required. The dog was intubated, and anaesthesia was maintained with inhalant isoflurane (Isoflurane; Fresenius Kabi Canada Ltd., Toronto, Ontario). A constant rate infusion of fentanyl (5–10 μg/kg/h BW, IV) was initiated. Peri‐ and intraoperative doses of cefazolin, (22 mg/kg BW, IV) (Cefazolin; Fresenius Kabi Canada Ltd., Toronto, Ontario) were administered every 90 min. The dog was maintained on mechanical ventilation throughout the thoracotomy.

**FIGURE 2:**
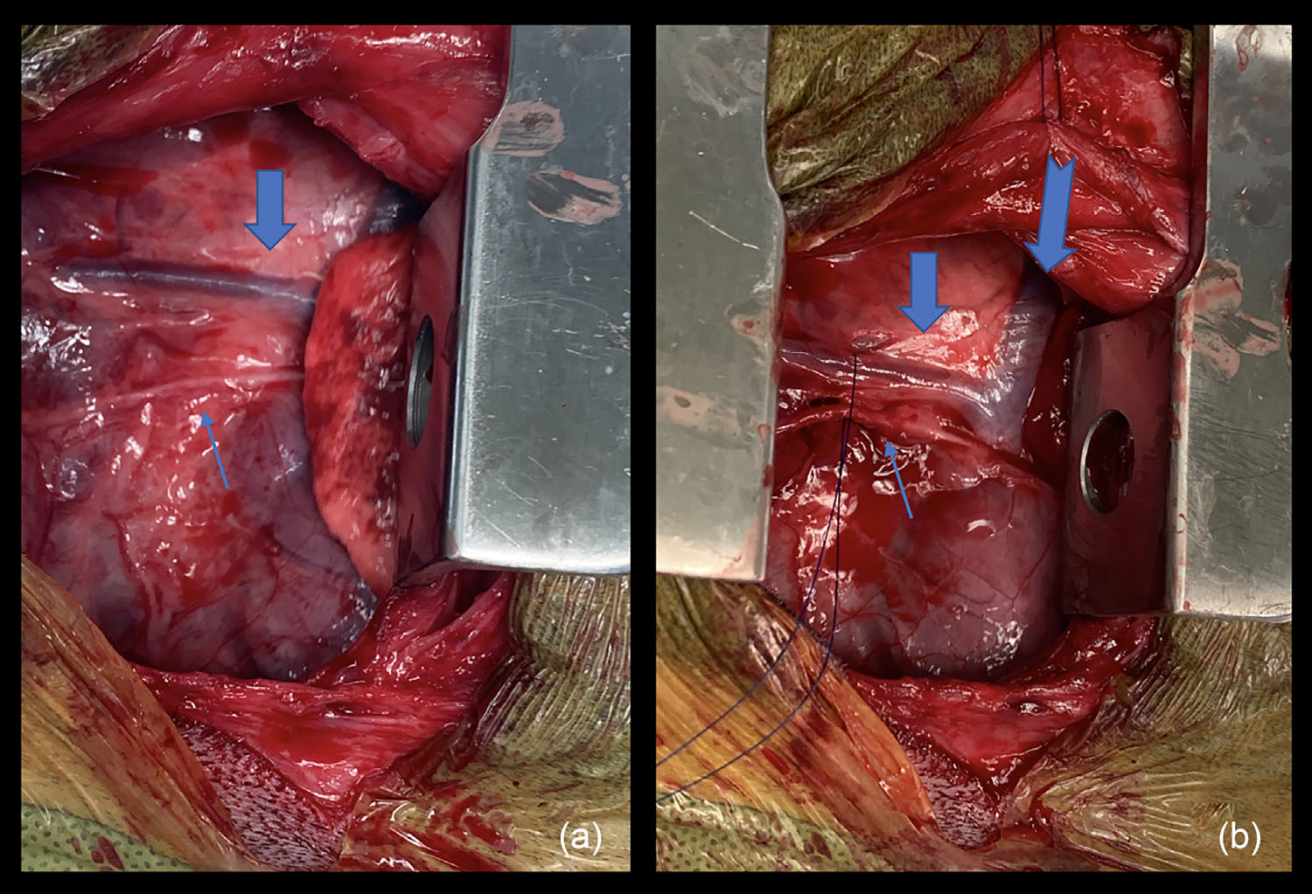
(a) Through a left fourth intercostal thoracotomy approach, the anomalous left vertebral vein (thick blue arrow) and the vagus nerve (thin blue arrow) are identified. (b) The anomalous left vertebral vein (thick and short blue arrow) and the vagus nerve (thin blue arrow) are encircled with a stay suture and the anomalous left azygos vein (long and thick blue arrow) is identified.

**FIGURE 3:**
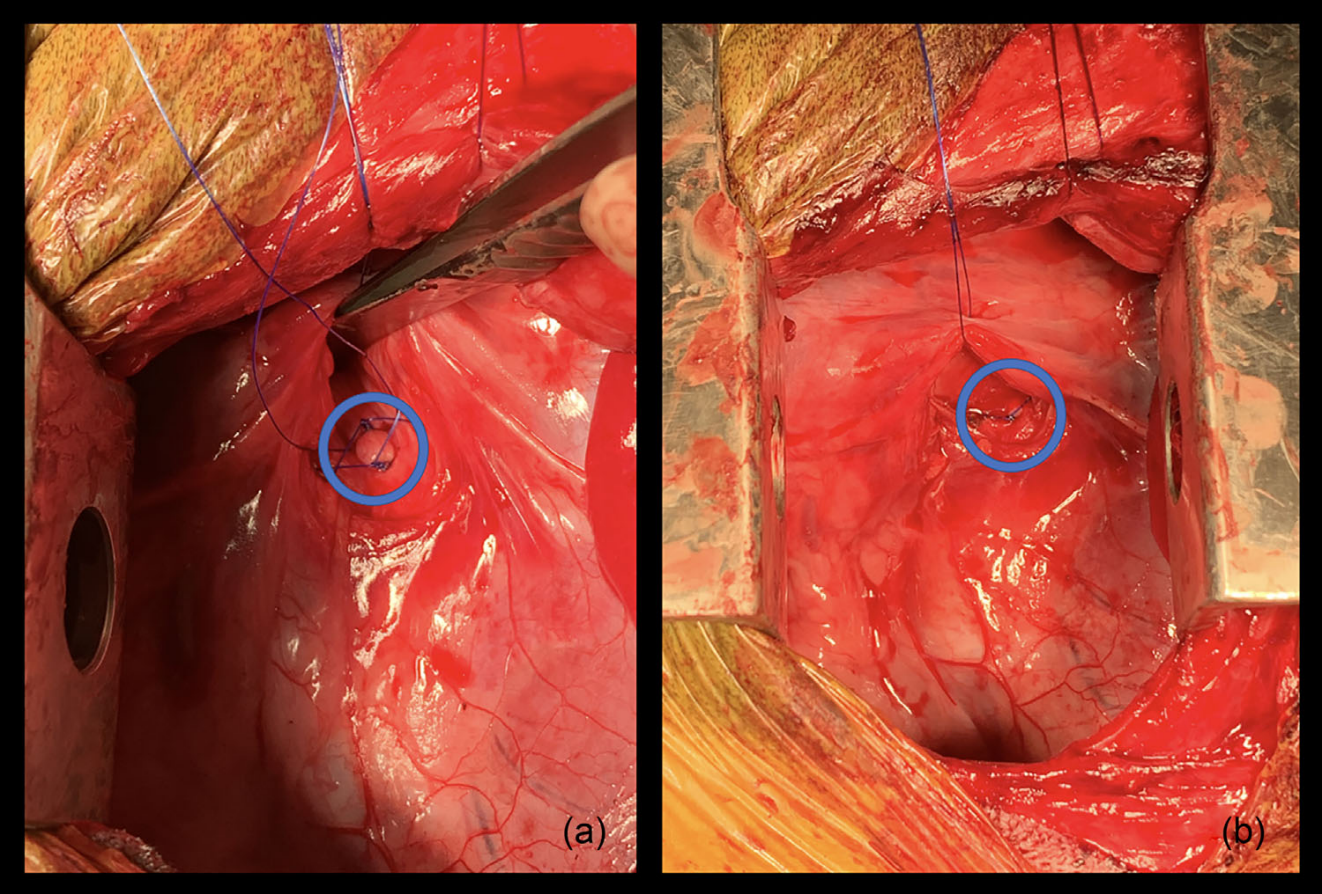
(a) The patent ductus arteriosus (PDA) has been double ligated with 3‐0 Prolene suture (blue circle). (b) The PDA has been transected and the ventral half of the PDA can be seen retracted towards the main pulmonary artery (blue circle). The dorsal half of the PDA retracted dorsally beneath the soft tissues and cannot be visualized.

A standard left fourth intercostal thoracotomy was performed. The left cranial lung lobe was reflected caudally. The vagus nerve and anomalous left vertebral vein were retracted dorsally with a stay suture of 3‐0 polydioxanone (PDS‐II; Ethicon Inc., Somerville, NJ) (Figure 2). The PDA was identified and the surrounding soft tissue was bluntly dissected with right angle forceps until the PDA was skeletonized. The PDA was approximately 1 cm in length and 0.5 cm in width. Turbulent blood flow was palpable at the junction between the PDA and the pulmonary artery. The PDA was double ligated with 3‐0 polypropylene suture (Prolene; Ethicon Inc.) and transected with Metzenbaum scissors between the ligatures (Figure 3). The transected portions of the PDA retracted from one another. An orogastric tube was passed into the oesophagus, resulting in further retraction of the transected portions of the PDA. The orogastric tube was removed and an endoscope was passed orally to visualize the oesophagus. A persistent area of extraluminal oesophageal compression was noted. An extraluminal fibrous band was transected using monopolar electrocautery. Esophagoscopy confirmed resolution of the oesophageal compression. The thorax was lavaged and a 14 gauge, thoracostomy tube (thoracostomy tube; MILA International Inc, Florence, Kentucky) was placed under visual guidance and secured to the skin with 3‐0 polypropylene suture (Prolene; Ethicon Inc.). The ribs were apposed with 2‐0 polydioxanone suture (PDS‐II; Ethicon Inc.) in a circumcostal pattern. The remainder of the thoracotomy approach was closed using a routine three‐layer closure. A 6‐in. wound diffusion catheter (Wound diffusion catheter 6″; MILA International Inc) was placed below the subcutaneous tissues, exiting dorsal and caudal to the thoracotomy incision and was secured to the skin with 3‐0 polypropylene suture (Prolene; Ethicon Inc.). The thoracostomy tube (thoracostomy tube; MILA International Inc.) was evacuated until negative pressure was achieved.

Post‐operative right lateral and ventrodorsal thoracic radiographs confirmed appropriate thoracostomy tube placement and resolution of the iatrogenic pneumothorax while also revealing a diffuse patchy interstitial coalescing to alveolar pulmonary pattern, most prominent in the right hemithorax; this was consistent with atelectasis or chronic aspiration pneumonia. Anaesthesia recovery proceeded without complication.

Post‐operative care included administration of intravenous fluids (Plasma‐Lyte; Baxter, Deerfield, Illinois) at a 5 mL/kg/h BW. Post‐operative pain management included a tapering constant rate infusion of fentanyl (3–5 μg/kg/h BW, IV) (Fentanyl; McKesson Canada, Saint‐Laurent, Quebec), bupivacaine (1.26 mg/kg BW; q6) (Sensorcaine; Sterimax Inc, Oakville, Ontario) administered into the wound diffusion catheter (Wound diffusion catheter 6″; MILA International Inc), gabapentin (8.4 mg/kg BW, PO; q8) (Teva Pharmaceutical Industries Ltd., Toronto, Ontario) and meloxicam (0.1 mg/kg BW, IV; q24) (Metacam; Boehringer Ingelheim, Animal Health Canada, Burlington, Ontario). Cefazolin (22 mg/kg BW, IV; q8) (Cefazolin, Fresenius Kabi Canada Ltd., Toronto, Ontario) was continued to treat presumptive aspiration pneumonia. Maropitant citrate (1 mg/kg BW, IV) (Cerenia, Zoetis Inc., Kalamazoo MI) was administered once immediately after recovery to reduce anaesthesia‐associated nausea. The thoracostomy tube was removed 24 h after surgery. The dog continued to be fed slurry of canned food in an upright position and remained upright for 15 min after eating. The palpable cervical oesophagus persisted after meals; however, there were no episodes of regurgitation observed in hospital post‐operatively.

The dog was discharged from hospital 2 days postoperatively with meloxicam (Meloxadin; Norbrook Laboratories Limited, Newry, Northern Ireland) (0.1 mg/kg PO q24h with food for 5 days), gabapentin (Gabapentin; Teva Canada) (8.4 mg/kg PO q12h for 3 days) and amoxicillin and clavulanic acid (Aventiclav; Norbrook Laboratories Limited, Newry, Northern Ireland) (13.75 mg/kg PO q12h). Antimicrobial therapy was recommended to be continued for 2 weeks beyond radiographic resolution of suspected aspiration pneumonia. Diet recommendations included a gradual transition from a liquid diet to kibble, accompanied with a transition from elevated feeding to non‐elevated feeding over a 6–8‐week period, if no regurgitation was occurring.

Twelve months post‐surgery, the dog weighed 26 kg, was in good body condition and was being fed a mixture of canned food and kibble from an elevated slow feeder. The owners reported no further regurgitation.

## 3 DISCUSSION

Previous retrospective studies have estimated the prevalence of congenital cardiovascular defects in dogs to be 1%, with PRAA representing 8% of all cardiovascular defects (Paterson, 1971). PRAA is the most common clinically relevant vascular ring anomaly, representing 95% of clinically affected animals (Buchanan, 2004). Vascular comorbidities with PRAA are not uncommon with reports of up to 44% of dogs with PRAA having at least one coexisting compressive arterial anomaly (Buchanan, 2004). PDA is a far less common comorbidity in dogs with PRAA, being identified concurrently in only 10%–14% of dogs (Buchanan, 2004; Schorn et al., 2021; vanGrundy, 1989). Although many reports of combined PRAA and PDA in both neonatal dogs and humans have identified the usual high‐grade continuous murmur of a PDA, the dog in this report lacked an audible heart murmur (Christiansen et al., 2007; Dundie et al., 2017; Holt et al., 2000; Reed & Bonasch, 1964; Salamat & Lyon, 2009; Saunders et al., 2013). Pre‐operatively identifying a PDA in dogs with PRAA is crucial as it may alter the surgical approach to reduce the risk of life‐threatening haemorrhage; the leading cause of operative death in cases of PDA (Birchard et al., 1990). Therefore, it is especially important that a PDA not be excluded based on lack of audible heart murmur.

Diagnostic evaluation for PRAA often begins with thoracic radiography; however, multiple diagnostic imaging modalities are beneficial in pre‐operative planning. CTA, while more invasive than radiography, can provide more detailed information about vascular comorbidities in animals with PRAA, including coexisting compressive anomalies (Buchanan, 2004). Despite its utility, a recent retrospective study evaluating dogs with PRAA from 1998 to 2015 found that only 7% of surgical interventions for PRAA used CTA for preoperative planning (Nucci et al., 2018). This likely underrepresents the overall use of CTA for vascular ring anomaly evaluation, as minimally invasive surgical (MIS) techniques for the treatment of PRAA have become more popular (Townsend et al., 2016). Thus, the use of advanced imaging to collect more anatomic information preoperatively is paramount to ensure correct selection of animals for these approaches (Townsend et al., 2016). Beyond, the use for determination of suitability for MIS techniques, advanced imaging further enhances the utility of other advanced pre‐surgical planning techniques such as 3D printing for these complex vascular anomalies (Dundie et al., 2017). Furthermore, echocardiography can complement CTA findings by confirming the presence of a concurrent PDA, such as in this case where an audible murmur was not identified and by evaluating the secondary cardiovascular effects of PDA preoperatively, such as presence of left ventricular and atrial dilation (Hamabe et al., 2015). Without the combined CTA and echocardiogram, a PDA was not suspected in this dog and may have led to life‐threatening haemorrhage had it been transected intraoperatively, without this knowledge.

Although both PRAA and PDA can be treated with minimally invasive surgery when occurring in isolation, occlusion or ligation of the PDA alone when occurring simultaneously with a PRAA would not resolve the vascular ring causing oesophageal compression and clinical signs for the affected animal (Gordon & Miller, 2005; Nucci et al., 2018; Townsend et al., 2016). Therefore, when PDA and PRAA occur simultaneously ligation and division of the PDA is required to resolve the compression from the vascular ring. Although it is not uncommon for the LA to be ligated during surgical treatment of PRAA in isolation to minimize the risk of haemorrhage, the uses of vessel sealing devices are commonly employed when PRAA is treated with MIS techniques (Nucci et al., 2018; Townsend et al., 2016). In a recent human case report, the uses of vessel sealing devices for division of PDA have been questioned due to the decreased amount of collagen present in the PDA relative to other vessels and the resultant significant haemorrhage that had been encountered during its use (Zamfir et al., 2007). Considering these factors, when a PDA is identified simultaneously with a PRAA, surgical approaches should be carefully considered. For this reason, a MIS approach was exchanged for an open thoracotomy with PDA ligation and division for the dog in this report.

## 4 CONCLUSION

This case is the first report of a dog with a combined PRAA and PDA, with the lack of an audible heart murmur. This case highlights the importance of using multiple diagnostic imaging modalities for preoperative surgical planning when a PRAA is suspected to best characterize the vascular anomaly. Specifically, the use of multiple imaging modalities may be required to identify a concurrent PDA as a PDA cannot be definitively ruled out by lack of audible murmur when occurring simultaneously with a PRAA.

## AUTHOR CONTRIBUTIONS

*Writing—original draft (lead); review and editing*: Marco T. Duguay. *Writing—review and editing*: Meagan A. Walker, Justyna Ostrowska, and Katie L. Hoddinott.

## CONFLICT OF INTEREST STATEMENT

The authors declare no conflict of interest.

5

### PEER REVIEW

The peer review history for this article is available at https://www.webofscience.com/api/gateway/wos/peer‐review/10.1002/vms3.1164.

## ETHICS STATEMENT

The authors confirm that the ethical policies of the journal, as noted on the journal's author guidelines page, have been adhered to. No ethical approval was required as this is a case report with no original research data.

## Data Availability

None.
